# Simultaneous in vivo detection of spectrally resolved glutamate, glutamine, and glutathione at 3 T with NAA‐aspartyl editing and echo‐time optimization

**DOI:** 10.1002/mrm.70076

**Published:** 2025-09-08

**Authors:** Li An, Sungtak Hong, Tara Turon, Adriana J. Pavletic, Christopher S. Johnson, Jun Shen

**Affiliations:** ^1^ National Institute of Mental Health National Institutes of Health Bethesda Maryland USA

**Keywords:** ^13^C, glutamate, glutamine, glutathione, NAA‐CH_2_

## Abstract

**Purpose:**

To achieve spectrally resolved in vivo detection of glutamate, glutamine, and glutathione at 3 T.

**Methods:**

Difference editing of N‐acetylaspartate CH_2_ protons (NAA‐CH_2_) combined with a new echo‐time (TE) optimization approach is introduced. Difference editing was used to detect NAA‐CH_2_ independently of NAA‐CH_3_, thereby eliminating systematic errors arising from constrained fitting of the entire NAA molecule. Numerical optimization of TE and TE_1_ minimized interference from highly dominant glutamate in glutamine detection in the ON/OFF sum spectrum. In vivo data were acquired from 6 healthy participants, including 2 who underwent oral administration of [U‐^13^C]glucose.

**Results:**

The NAA‐aspartyl‐edited, cleaned‐up in vivo spectrum showed distinct separation of glutamate, glutamine, and glutathione peaks at 3 T, facilitating spectral quantification and clinical applications. The post‐^13^C proton MR‐spectroscopy spectra clearly demonstrated the dynamic ^13^C‐labeling of glutamate C4 following oral [U‐^13^C]glucose intake.

**Conclusion:**

This technique enables simultaneous spectral resolution of glutamate, glutamine, and glutathione peaks at 3 T using difference editing of NAA‐CH_2_ and an optimized TE of 85 ms. Additionally, it demonstrates, for the first time, the feasibility of measuring ^13^C turnovers of spectrally resolved glutamate at 3 T with the high sensitivity and spatial resolution of proton MR spectroscopy.

## 1 INTRODUCTION

Glutamate (Glu) is the primary excitatory neurotransmitter in the central nervous system, whereas astroglial glutamine (Gln) is metabolically linked to neuronal Glu through the Glu‐Gln cycle.^1^, ^2^, ^3^ Glutathione (GSH), a major antioxidant, serves as a marker of redox state.^4^ Altered levels of Glu, Gln, and GSH have been implicated in many neuropsychiatric disorders, including epilepsy, schizophrenia, bipolar disorder, and major depressive disorder.^5^, ^6^, ^7^, ^8^, ^9^, ^10^, ^11^

**FIGURE 1:**
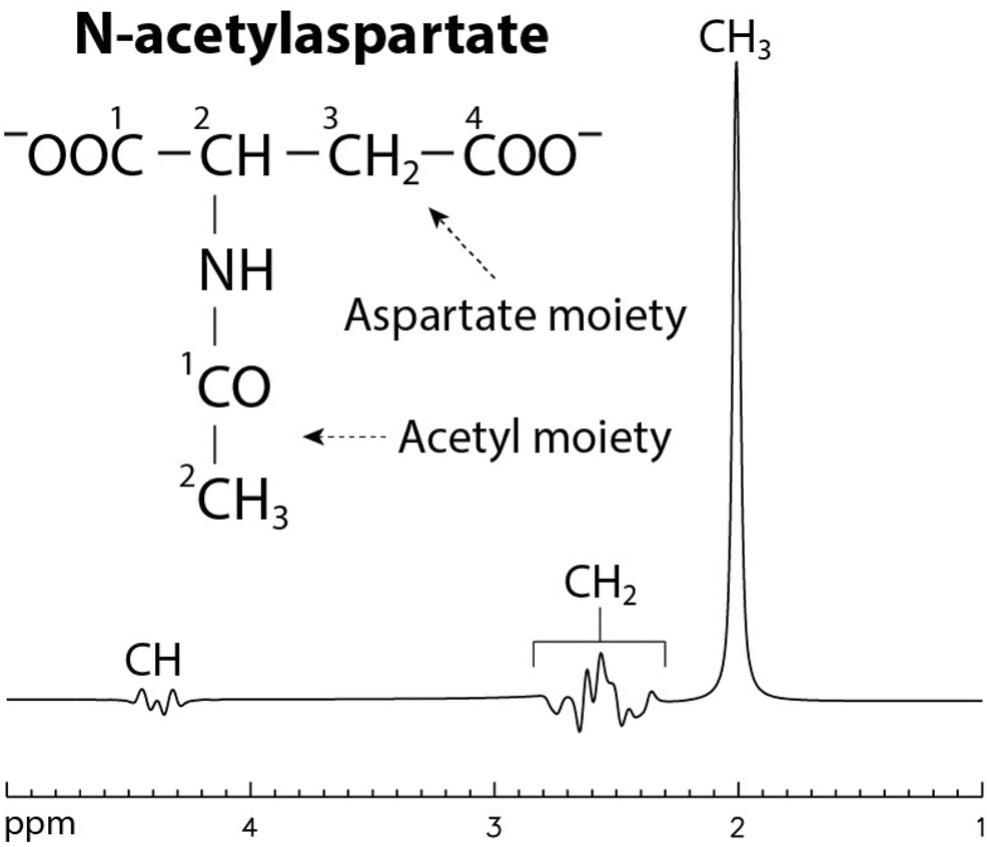
Structural formula and numerically computed spectrum of N‐acetylaspartate. A point‐resolved spectroscopy sequence with echo time = 85 ms was used for the numerical simulation.

In vivo detection of Glu, Gln, and GSH using MR spectroscopy (MRS) at field strengths of 3 T or lower is often hampered by spectral overlap, including (i) interference from the aspartate moiety of N‐acetylaspartate (NAA‐CH_2_) (see Figure 1) with the detection of Gln and GSH, and (ii) interference from dominant Glu in Gln detection. For the first issue, NAA‐CH_2_ is typically quantified through spectral modeling alongside other metabolites, assuming the T_2_‐weighted concentration of NAA‐CH_2_ is identical to that of the NAA acetyl moiety (NAA‐CH_3_). However, studies have shown that the two moieties of NAA have significantly different T_2_ relaxation times at 3 T.^12^, ^13^ For example, T_2_ values of 258 ms for NAA‐CH_3_ and 222 ms for NAA‐CH_2_ have been reported.^12^ Consequently, constrained fitting of the entire NAA molecule is logically incorrect and introduces systematic errors in the quantification of NAA‐CH_2_, which in turn lead to systematic errors in the measurement of Gln and GSH due to spectral overlap. For the second issue, various MRS techniques have been developed to improve the spectral resolution of Glu and Gln.^14^, ^15^, ^16^, ^17^, ^18^, ^19^ For example, in Zhang and Shen,^19^ a two‐dimensional J‐coupled point‐resolved spectroscopy (PRESS) data set was acquired at 3 T for spectral isolation of Gln from the highly dominant and overlapping Glu signal.

This study introduces difference editing of NAA‐CH_2_ combined with a novel TE optimization approach to enable simultaneous and spectrally resolved detection of Glu, Gln, and GSH at 3 T. The two spectral overlap issues described are isolated and resolved. To address the first issue, NAA‐CH_2_ is quantified using difference editing, independently of NAA‐CH_3_, and its signal contribution to the ON/OFF sum spectrum ([ON + OFF]/2) is computed and subtracted along with the significantly weaker aspartate (Asp) and N‐acetylaspartylglutamate (NAAG) signals. To address the second issue, the TE and TE_1_ values for the difference editing sequence are numerically optimized to minimize interference from the highly dominant Glu signal in the detection of Gln while maintaining high signal intensities for Glu, Gln, and GSH in the sum spectrum. This technique enables the acquisition of in vivo spectra with distinct separation of Glu, Gln, and GSH peaks in a single 3T experiment. Additionally, ^1^H MRS with NAA‐CH_2_ editing at TE = 85 ms demonstrates the feasibility of noninvasively monitoring ^13^C labeling of spectrally resolved Glu in the human brain at 3 T following [U‐^13^C]glucose administration.

## 2 METHODS

### 2.1 NAA‐CH_2_ editing

The proposed pulse sequence incorporated a single 180° editing pulse^20^ between the two 180° slice‐selective refocusing pulses of a PRESS sequence^21^ (see Figure S1). The slice‐selective excitation pulse was an asymmetric amplitude‐modulated pulse with a duration of 4.5 ms and a full‐width half‐maximum bandwidth of 3.1 kHz, and a rephase factor of 0.167.^22^ The symmetric slice‐selective refocusing pulses were amplitude‐modulated with a duration of 8.0 ms and a full‐width half‐maximum bandwidth of 2.0 kHz.

**FIGURE 2:**
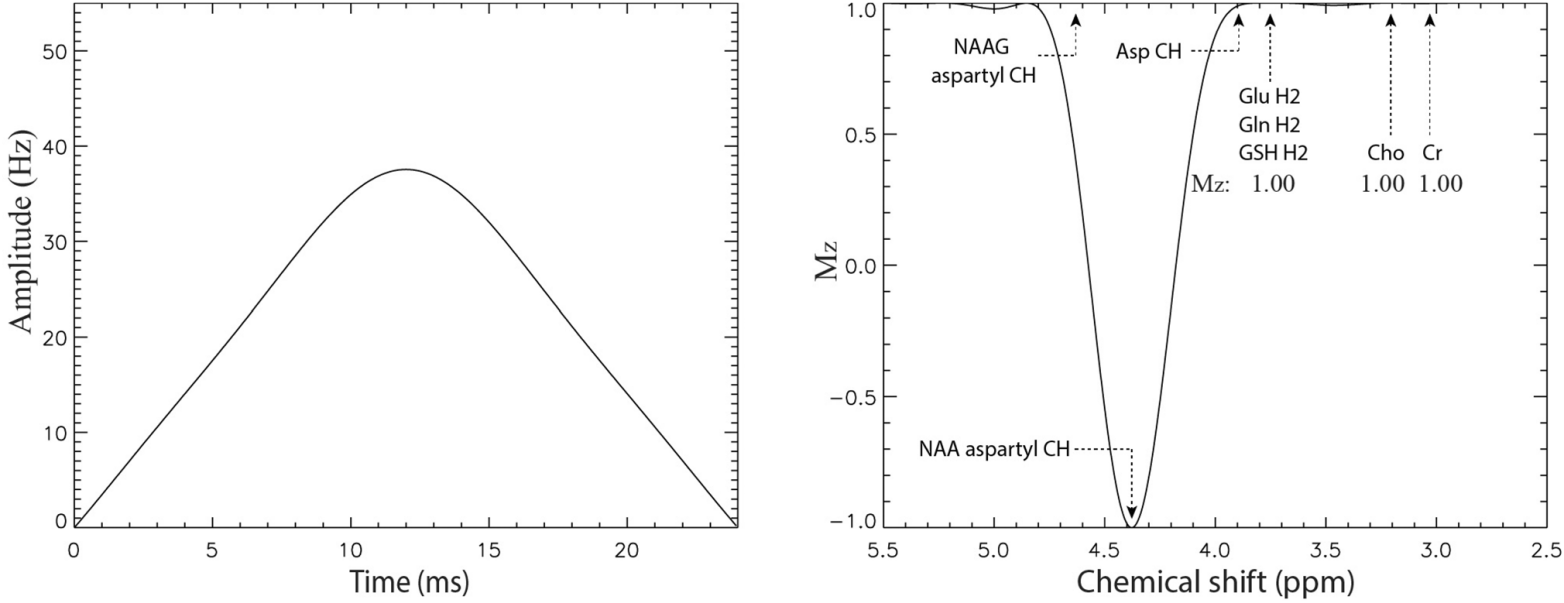
Pulse amplitude (*left*) and frequency response (*right*) of a custom dual‐band pulse. Pulse duration was 24 ms. The value of M_z_ is expressed relative to the equilibrium magnetization M_0_. Asp, aspartate; Cho, choline; Cr, creatine; Glu, glutamate; Gln, glutamine; GSH, glutathione; NAA, N‐acetylaspartate; NAAG, N‐acetylaspartylglutamate.

The editing pulse was constructed using a superposition of two symmetrically truncated Gaussians with the same duration of 24 ms (see Procedure S1 and Table S1).^23^, ^24^ The first Gaussian band was centered at 4.382 ppm to invert the NAA‐CH proton, whereas the second Gaussian band, with an amplitude 6.3% of the first, was centered at 3.755 ppm to eliminate perturbations on the α‐protons of Glu, Gln, and glutamyl‐GSH (see Figure S2). As shown in Figure 2, the vector‐summed dual‐band pulse successfully avoided perturbing the resonances of Glu, Gln, and glutamyl‐GSH. Consequently, the editing pulse had no effect on the Glu, Gln, and glutamyl‐GSH H4 intensities. In addition to inverting the NAA‐CH proton at 4.38 ppm, the editing pulse also partially inverted the aspartyl CH proton of NAAG at 4.63 ppm.

### 2.2 TE optimization to minimize Glu interference in Gln detection

To minimize interference from Glu in the detection of Gln, TE optimization was conducted using density matrix simulations with high spatial resolution.^25^, ^26^ These simulations computed the ON/OFF sum spectrum of Glu, Gln, and GSH across 10 TE values, ranging from 75 to 120 ms in 5‐ms increments. Shorter TE values were excluded to accommodate the editing pulse. After Bloch‐Siegert shift correction,^27^ the upfield portion (< 3.9 ppm) of the sum spectrum, along with its parent ON and OFF spectra, remained practically unchanged, as the editing pulse did not perturb the resonance signals of Glu, Gln, or glutamyl‐GSH (Figure 2). In these simulations, concentration ratios of Glu, Gln, and GSH were set to 1 : 0.30 : 0.35,^17^ with T_2_ values of 185, 145, and 89 ms, respectively.^19^, ^28^ A 3.5‐Hz line broadening with a Voigt lineshape (50% Lorentzian, 50% Gaussian) was applied to account for line broadening due to B_0_ inhomogeneity. To quantify interference from the dominant Glu signal in the detection of Gln, a Glu interference metric was defined as the ratio of the sum of the absolute values of the real‐valued Glu spectrum to that of the Gln spectrum within the same 2.38–2.50 ppm range. For each TE value, spectra were computed using all practically feasible TE_1_ values in 1‐ms intervals, and the TE_1_ that minimized the Glu interference metric was selected. Among the 10 TE values examined, the TE associated with the smallest Glu interference metric was identified as the optimal TE. The peak heights of Glu, Gln, and GSH at each TE were also calculated to ensure that the signal intensities of these metabolites were high at the optimal TE relative to the other TEs.

### 2.3 In vivo experiments

Six healthy participants (5 females and 1 male; age = 32 ± 16 years) were recruited for this study. MRS scans were conducted using a Siemens Skyra 3T scanner equipped with a single‐channel transmit and 32‐channel receive coil. Written informed consent was obtained from all participants before the study, following procedures approved by the Institutional Review Board of the National Institute of Mental Health (NCT01266577, NCT00109174). All experiments were conducted in accordance with the guidelines and regulations of the National Institutes of Health MRI Research Facility. A three‐dimensional T_1_‐weighted magnetization‐prepared rapid gradient‐echo image was acquired with repetition time (TR) = 3 s, TE = 3.0 ms, and spatial resolution = 1 × 1 × 1 mm^3^. A 3 × 3 × 2 cm^3^ MRS voxel was placed in the anterior cingulate cortex with an in vivo water linewidth of 6.4 ± 0.5 Hz.

The numerically optimized pulse sequence (TE = 85 ms, TE_1_ = 26 ms, T_d_ = 21 ms, TR = 2.5 s) was used to acquire MRS data, where T_d_ represents the center‐to‐center time delay between the first slice‐selective refocusing pulse and the editing pulse (see Figure S1). Water suppression was achieved using seven variable power radiofrequency pulses (sinc‐Gauss pulse, duration = 26 ms, bandwidth = 105 Hz) with optimized relaxation delays. ON and OFF acquisitions were alternated for a total of 70 pairs, resulting in a scan time of 6 min.

The 32‐channel free induction decay (FID) data were combined into optimally weighted composite FIDs using the generalized least squares method,^29^ with coil sensitivities derived from unsuppressed water signals acquired with two acquisitions. These unsuppressed water signals were also used to correct phase errors in the combined FIDs caused by zero‐order eddy currents.^30^ The combined FIDs were Fourier‐transformed into the frequency domain to generate spectra for individual acquisitions. The Bloch‐Siegert phase shift in each ON spectrum, caused by the single editing pulse, was numerically corrected.^27^

Frequency drift correction was applied simultaneously to each pair of ON and OFF spectra to avoid introducing subtraction errors in the difference spectrum. The frequency drift for each ON/OFF pair was determined by fitting the NAA, creatine (Cr), and choline (Cho) singlets in the sum spectrum and was subsequently corrected in the individual ON and OFF spectra. The corrected ON spectra were then averaged to produce a single ON spectrum for the scan, whereas the OFF spectra were averaged to yield a single OFF spectrum. To ensure complete phase alignment, any small relative phase difference between the ON and OFF spectra was eliminated by fitting the two spectra within the parts‐per‐million (ppm) ranges encompassing the NAA, Cr, and Cho singlets. Finally, a sum spectrum and a difference spectrum for the scan were computed from these processed ON and OFF spectra.

For computing metabolite basis functions, chemical shifts and coupling constants were obtained from Kaiser et al.^31^ for γ‐aminobutyric acid (GABA), de Graaf^32^ for Glu, Choi et al.^33^ for GSH, and Govind^34^ for acetate, NAA, NAAG, Gln, Asp, Cr, phosphocreatine, phosphocholine, glycerophosphocholine, taurine, myo‐inositol, and scyllo‐inositol.

The effects of frequency drifts on the metabolite basis functions including NAAG were also corrected.^26^, ^35^ Frequency drift altered the effective frequency of the editing pulse during the ON acquisition. To account for this, basis spectra for the ON acquisition were numerically computed for 17 integer frequency drift values within ±8 Hz. The frequency drifts previously determined from fitting the in vivo spectra were discretized into these 17 integer values. Based on the discretized drifts for the 70 in vivo ON/OFF pairs, 70 corresponding ON basis spectra were generated for each metabolite. These were then averaged to produce a single, drift‐corrected ON basis spectrum. Finally, the basis sum and basis difference spectra were derived. Further spectral simplification and quantification were performed by fitting the difference spectrum and sum spectra following a four‐step procedure:

1. The in vivo sum spectrum was fitted with the basis sum spectra of all metabolites and a cubic spline baseline in the range of 1.8–3.4 ppm. The zero‐order phase determined from the fit was removed from both the in vivo sum and difference spectra.
2. Using the NAAG concentration obtained from Step 1, the NAAG signal in the in vivo difference spectrum was numerically computed and subtracted. The resulting difference spectrum was then fitted with the basis difference spectrum of NAA‐CH_2_ in the range of 2.20–2.87 ppm to determine the NAA‐CH_2_ concentration.
3. Using the NAA‐CH_2_ concentration obtained from Step 2, the NAA‐CH_2_ signal in the in vivo sum spectrum was numerically computed and subtracted, along with the fitted NAAG signal from Step 1. The resulting sum spectrum was fitted in the range of 2.65–2.87 ppm with the basis sum spectrum of Asp to quantify Asp, and the fitted Asp signal was subsequently subtracted.
4. The cleaned‐up in vivo sum spectrum, with the modeled NAA‐CH_2_, NAAG, and Asp signals subtracted, was fitted in the range of 1.8–3.4 ppm to quantify Glu, Gln, GSH, and other remaining metabolites.

**FIGURE 3:**
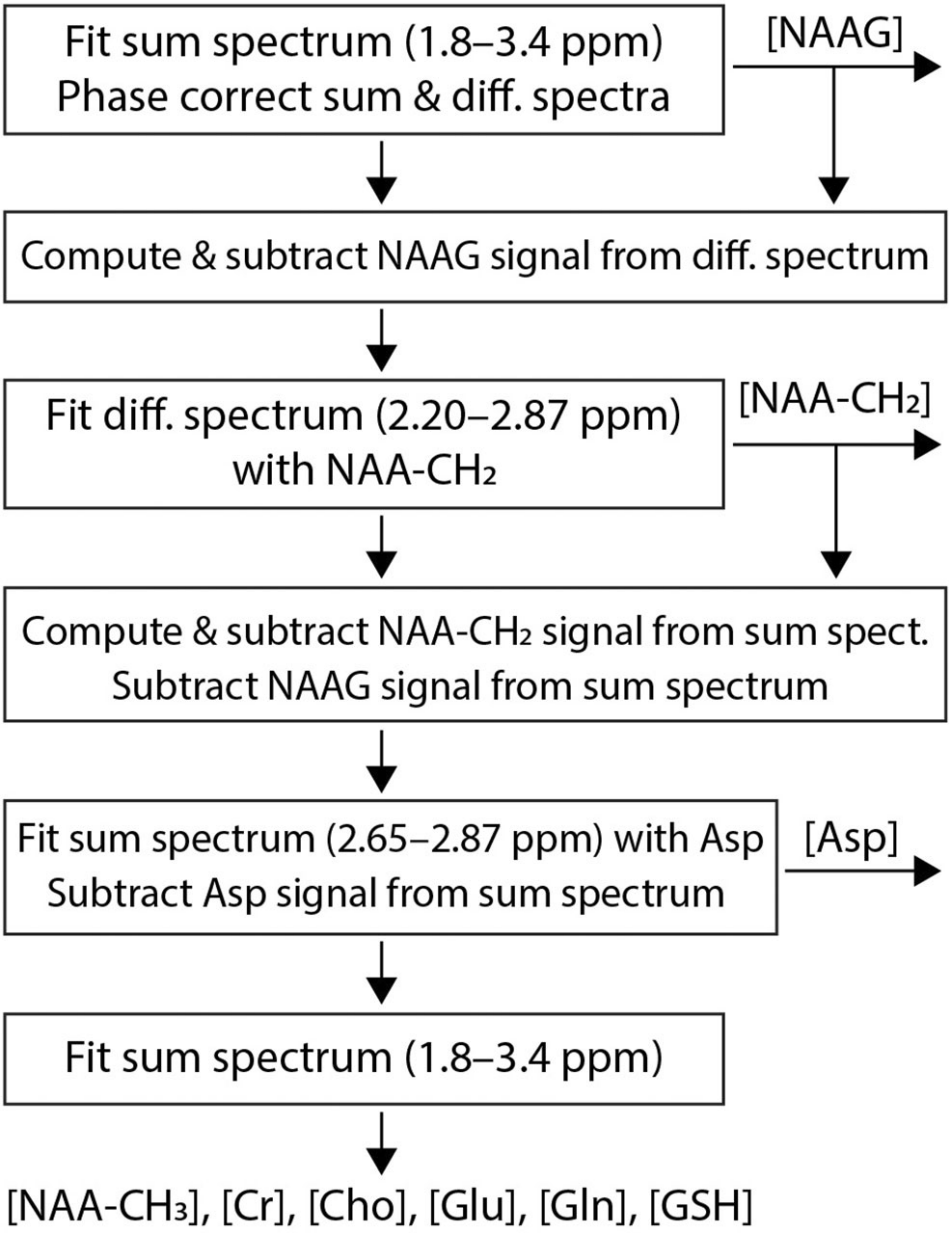
Flowchart of spectral simplification and quantification. Asp, aspartate; Cho, choline; Cr, creatine; Glu, glutamate; Gln, glutamine; GSH, glutathione; NAA, N‐acetylaspartate; NAA‐CH_2_, N‐acetylaspartate CH_2_ protons; NAAG, N‐acetylaspartylglutamate.

A flowchart illustrating this procedure is shown in Figure 3.

### 2.4 Comparison with constrained fitting of the entire NAA molecule

Monte Carlo analysis was conducted to compare the proposed technique with a conventional method that quantifies metabolite concentrations with constrained fitting of the entire NAA molecule. A pair of noise‐free ON and OFF FIDs were simulated by combining basis functions based on in vivo concentrations, linewidths, and lineshape of the metabolite resonance signals. T_2_ relaxation decays were applied to the basis functions using T_2_ = 258 ms for NAA‐CH_3_ and T_2_ = 222 ms for NAA‐CH_2_.^12^ White Gaussian noise, matching the in vivo noise level, was added to the noise‐free FIDs. The resulting FIDs were processed to generate simulated spectra for both the conventional method and the proposed technique, which were then quantified using their respective methods. This process of adding noise to the noise‐free FIDs, generating the noise‐added spectra, and quantifying them was repeated 1000 times, with each iteration using a different realization of random noise at the same noise level.

### 2.5 Glu turnover

Two of the 6 healthy participants underwent scanning with oral administration of [U‐^13^C]glucose after overnight fasting. Following a pre‐^13^C MRS scan, the participants exited the scanner and drank 20% wt/wt 99% enriched [U‐^13^C]glucose solution at a dosage of 0.75 g [U‐^13^C]glucose per kilogram of body weight, following procedures described in our previous study of carbonic anhydrase‐catalyzed ^13^C magnetization transfer.^36^ After a rest period, the participants reentered the scanner for post‐^13^C MRS scans.

A pre‐^13^C spectrum and a series of post‐^13^C spectra were generated from the acquired data, each representing an ON/OFF sum spectrum. Due to ^1^H‐^13^C coupling, the parent Glu and Gln H4 peaks in these spectra decreased after the intake of [U‐^13^C]glucose. Consequently, the differences between the pre‐^13^C spectrum and the post‐^13^C spectra revealed changes in the Glu and Gln H4 signals caused by ^13^C labeling of Glu and Gln. To account for the linewidth and lineshape differences between the pre‐^13^C spectrum and each post‐^13^C spectrum, the following steps were taken: First, the pre‐^13^C spectrum was generated without line broadening, whereas the post‐^13^C spectra were generated with 2‐Hz line broadening using a Voigt lineshape (50% Lorentzian, 50% Gaussian). Then, the pre‐^13^C spectrum was fitted to each post‐^13^C spectrum within the ppm ranges encompassing the NAA, Cr, and Cho singlets, during which the pre‐^13^C spectrum was line‐broadened with a Voigt lineshape to best match the post‐^13^C spectrum. Finally, the difference spectra between the broadened pre‐^13^C spectrum and the post‐^13^C spectra were numerically computed.

## 3 RESULTS

The amplitude profile and frequency response of the custom dual‐band editing pulse are shown in Figure 2. The frequency response demonstrated that the dual‐band pulse effectively avoided perturbing the α‐protons of Glu, Gln, and glutamyl‐GSH at about 3.75 ppm (M_z_ = 1.00). Without the second Gaussian band at 3.755 ppm, the α‐protons of Glu, Gln, and glutamyl‐GSH would be noticeably perturbed (M_z_ = 0.98; see Figure S2).

**FIGURE 4:**
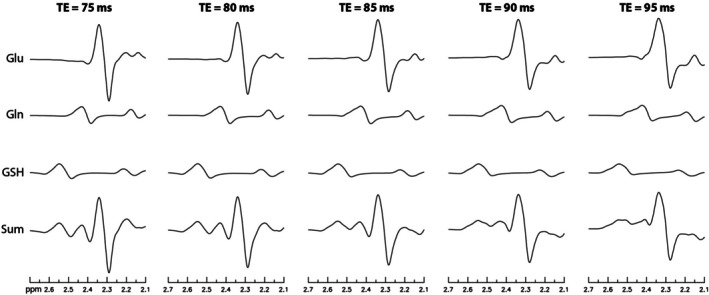
Numerically computed spectra of glutamate (Glu), glutamine (Gln), and glutathione (GSH) at five different echo‐time (TE) values, with T_2_ decay considered. The concentration ratios of Glu, Gln, and GSH were set to 1: 0.30: 0.35, with T_2_ values of 185, 145, and 89 ms, respectively. Line broadening due to B_0_ inhomogeneity was set to 3.5 Hz, corresponding to a 7.0‐Hz water linewidth (assuming a water T_2_ of 90 ms).

Numerically computed spectra for five different TE values, with T_2_ decay considered, are presented in Figure 4. Each spectrum in Figure 4 represents an ON/OFF sum spectrum, which, with Bloch‐Siegert shift correction, was identical to the individual ON and OFF spectra within the displayed ppm range, as the editing pulse applied at 4.38 ppm avoided the resonances of Glu, Gln, and glutamyl‐GSH. At TE = 80 and 85 ms, the Glu peak appeared sharp with a flat downfield shoulder, minimizing interference with the resonance signal of Gln. Beginning at TE = 90 ms, the Glu peak increasingly overlapped the Gln peak as TE increased. By TE = 95 ms, the Gln peak was largely obscured by the Glu peak. This overlap became progressively more severe from TE = 100 to 120 ms (not shown in Figure 4).

The values of the Glu interference metric for three levels of line broadening and five TEs are provided in Table 1. Line broadenings due to B_0_ inhomogeneity (LB_B0_) in the range of 2.5–4.5 Hz correspond to water linewidths (LW_w_) of 6.0–8.0 Hz, which represent typical B_0_ shimming achievable for in vivo MRS experiments at 3 T with high‐order shimming. For all three line broadenings, TE = 85 ms yielded the lowest Glu interference metric among all TEs, indicating that it was optimal for minimizing Glu interference in Gln detection. The second‐best TE was 80 ms, which consistently produced the second‐lowest Glu interference metric across all three line broadenings. However, the Glu and Gln peak heights at TE = 80 ms were significantly lower than those at TE = 85 ms. These results confirmed that TE = 85 was the best choice for minimizing Glu interference in Gln detection while preserving high signal intensities for Glu, Gln, and GSH.

**TABLE 1:** TE optimization for minimizing Glu interference in Gln detection.

LB_B0_ (Hz)	LW_w_ (Hz)	TE (ms)	Glu interference metric (%)	Peak heights (a.u.)		
				Glu	Gln	GSH
2.5	6.0	75	59.9	8.25	2.03	1.94
		80	23.7	8.65	2.07	1.94
		85	21.7	9.04	2.10	1.88
		90	26.1	8.95	2.20	1.87
		95	39.2	9.09	2.20	1.83
3.5	7.0	75	61.2	6.83	1.76	1.74
		80	22.8	7.31	1.81	1.75
		85	20.3	7.81	1.86	1.71
		90	28.9	7.84	1.98	1.72
		95	42.1	8.08	2.01	1.70
4.5	8.0	75	63.2	5.66	1.55	1.55
		80	22.9	6.21	1.61	1.58
		85	20.5	6.78	1.67	1.56
		90	34.3	6.93	1.79	1.59
		95	48.5	7.29	1.83	1.59

*Note*: Three levels of line broadening due to B_0_ inhomogeneity (LB_B0_) are listed, along with the corresponding water linewidths (LW_w_) calculated assuming a water T_2_ of 90 ms. The Glu interference metric was defined as the ratio of the sum of the absolute values of the real‐valued Glu spectrum to that of the Gln spectrum within the same 2.38–2.50‐ppm range. T_2_ relaxation effects were accounted for using T_2_ values of 185 ms for Glu, 145 ms for Gln, and 89 ms for GSH.

Abbreviations: Gln, glutamine; Glu, glutamate; GSH, glutathione; TE, echo time.

**FIGURE 5:**
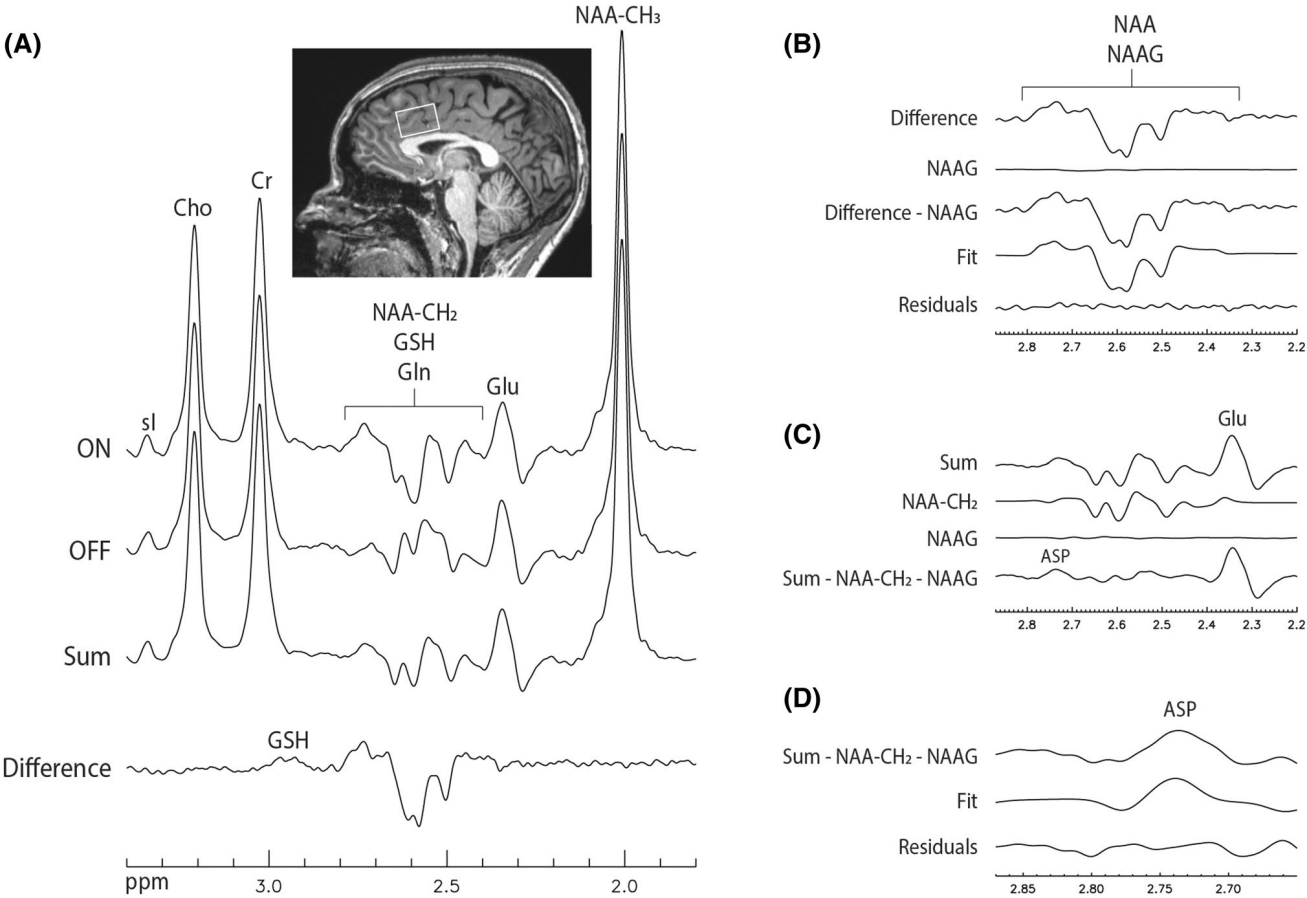
In vivo spectra acquired from the anterior cingulate cortex of a healthy participant using the proposed N‐acetylaspartate (NAA)–aspartyl editing technique, along with the fitting results for determining NAA‐CH_2_ and aspartate (Asp). No line broadening was applied. The NAA‐aspartyl editing pulse had a duration of 24 ms and was positioned 21 ms (center‐to‐center) after the first slice‐selective refocusing pulse (T_d_ = 21 ms). Voxel size = 3 × 3 × 2 cm^3^; echo time (TE) = 85 ms; TE_1_ = 26 ms; spectral width = 5000 Hz; number of data points = 2048; repetition time = 2.5 s; number of ON/OFF pairs = 70; total scan time = 6 min. (A) The acquired ON and OFF spectra and their sum and difference. (B) Quantification of NAA‐CH_2_ by fitting the difference spectrum in the 2.20–2.87‐ppm range after subtracting the modeled N‐acetylaspartylglutamate (NAAG) signal. (C) Subtraction of both the modeled NAA‐CH_2_ and NAAG signals from the sum spectrum. (D) Quantification of Asp by fitting the sum spectrum in the 2.65–2.87‐ppm range after subtracting the modeled NAA‐CH_2_ and NAAG signals. Cho, choline; Cr, creatine; Gln, glutamine; Glu, glutamate; GSH, glutathione.

**FIGURE 6:**
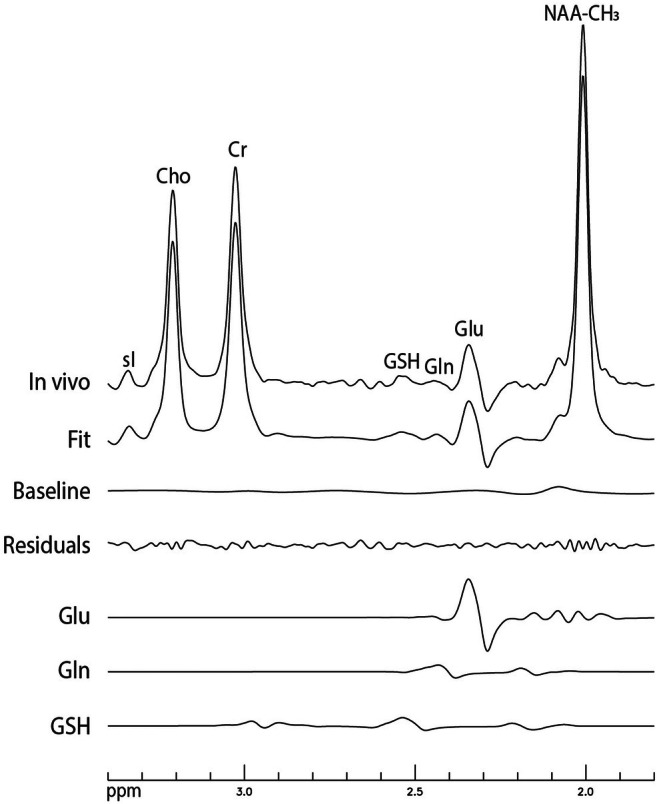
Final fitting results for determining the major metabolites in the participant's data shown in Figure 5. No line broadening was applied. The in vivo spectrum represents the cleaned‐up sum spectrum after subtracting the modeled N‐acetylaspartate CH_2_ protons (NAA‐CH_2_), acetylaspartylglutamate (NAAG), and aspartate (Asp) signals. Gln, glutamine; Glu, glutamate; GSH, glutathione.

In vivo spectra acquired using the proposed technique from a single participant, along with the fitting results, are presented in Figures 5 and 6. The ON and OFF spectra, as well as the sum and difference spectra derived from them, are displayed in Figure 5A. In the difference spectrum (bottom trace of Figure 5A), the Glu and Gln resonances were fully eliminated, confirming the validity of our fitting procedure described in Section 2 and the spectral selectivity of the editing pulse shown in Figure 2.

The difference spectrum in the range of 2.20–2.87 ppm, shown in the top trace of Figure 5B, contained only the NAA‐CH_2_ and NAAG signals. As described in Step 2 of the spectral simplification and quantification process, the numerically computed NAAG signal (second trace of Figure 5B) was subtracted from the difference spectrum. The resulting new difference spectrum (third trace of Figure 5B) was then fitted with the basis difference spectrum of NAA‐CH_2_ (fourth trace of Figure 5B) to quantify NAA‐CH_2_. The fitting residuals (bottom trace of Figure 5B) were minimized.

The sum spectrum in the range of 2.20–2.87 ppm is shown in the top trace of Figure 5C. The NAA‐CH_2_ signal (second trace of Figure 5C), numerically computed based on its concentration obtained in Step 2, and the NAAG signal (third trace of Figure 5C) obtained from the fitting in Step 1, were subtracted from the sum spectrum, yielding a sum spectrum without NAA‐CH_2_ or NAAG signals (bottom trace of Figure 5C). This sum spectrum only contained the Asp signal in the range of 2.65–2.87 ppm (top row of Figure 5D). It was therefore fitted with the basis sum spectrum of Asp (second trace of Figure 5D) to quantify Asp (Step 3).

After subtracting the modeled NAA‐CH_2_, NAAG, and Asp signals, the cleaned‐up in vivo sum spectrum (top trace of Figure 6) was fitted over the range of 1.8–3.4 ppm to quantify Glu, Gln, GSH, and the remaining metabolites. This cleaned‐up in vivo spectrum revealed clearly separated peaks for Glu, Gln, and GSH, with minimal residuals in the fit (Step 4).

Table 2 lists metabolite concentrations in the anterior cingulate cortex of 6 healthy participants. The Glu, Gln, and GSH concentrations were found to be 11.4 ± 1.3, 2.8 ± 0.8, and 3.9 ± 0.5 mM, respectively, with low Cramer‐Rao lower bounds (CRLBs) of 2.0% ± 0.4%, 6.8% ± 2.3%, and 5.2% ± 1.2%, respectively. The ratio between the raw concentrations of NAA‐CH_2_ and NAA‐CH_3_, obtained directly from spectral fitting, was 0.95 ± 0.03, significantly below 1 (one‐sample, one‐tailed t‐test; *p* = 0.01).

**TABLE 2:** Metabolite concentrations measured from the anterior cingulate cortex of healthy participants (*n* = 6) at 3 T.

	Concentration (mM)	CRLB (%)
Cr	9.4 ± 0.9	1.5 ± 0.3
Cho	2.5 ± 0.4	1.4 ± 0.3
^a^NAA‐CH_3_	11.9 ± 0.6	1.6 ± 0.4
^a^NAA‐CH_2_	11.9 ± 0.8	1.8 ± 0.3
Asp	2.2 ± 0.3	6.5 ± 1.1
NAAG	0.8 ± 0.2	7.4 ± 3.0
Glu	11.4 ± 1.3	2.0 ± 0.4
Gln	2.8 ± 0.8	6.8 ± 2.3
GSH	3.9 ± 0.5	5.2 ± 1.2

*Note*: The voxel composition was 51.3% ± 2.4% gray matter, 43.9% ± 2.3% white matter, and 4.7% ± 3.0% cerebrospinal fluid.

Abbreviations: Asp, aspartate; Cho, choline; Cr, creatine; CRLB, Cramer‐Rao lower bounds; Gln, glutamine; Glu, glutamate; GSH, glutathione; NAA, N‐acetylaspartate; NAAG, N‐acetylaspartylglutamate.

^a^ The ratio between the T_2_‐weighted concentrations of NAA‐CH_2_ and NAA‐CH_3_, obtained directly from spectral fitting, was 0.95 ± 0.03, significantly below 1 (one‐sample, one‐tailed *t*‐test, *p* = 0.01).

Monte Carlo analysis (Table 3) showed that constrained fitting of the entire NAA molecule resulted in substantial errors for Gln (3.0%) and GSH (9.4%). The proposed technique effectively eliminated these systematic errors.

**TABLE 3:** Monte Carlo analysis comparing constrained fitting of the entire NAA molecule and NAA aspartyl editing.

		Constrained fitting			NAA‐aspartyl editing		
	True value	Mean	Error (%)	CV (%)	Mean	Error (%)	CV (%)
Cr	9.40	9.40	0.0	1.5	9.41	0.1	1.5
Cho	2.50	2.50	0.0	1.6	2.50	0.1	1.6
NAA‐CH_3_	11.9	11.9	0.4	1.8	11.9	0.1	1.8
NAA‐CH_2_	11.9	11.9	0.4	1.8	11.9	0.0	2.2
Asp	2.20	2.21	0.3	7.8	2.20	0.1	8.7
NAAG	0.80	0.77	4.2	8.6	0.80	0.3	8.7
Glu	11.4	11.4	0.1	2.0	11.4	0.2	2.0
Gln	2.80	2.88	3.0	7.1	2.81	0.2	7.6
GSH	3.90	3.53	9.4	6.1	3.91	0.2	6.4

Abbreviations: Abbreviations: Asp, aspartate; Cho, choline; Cr, creatine; CRLB, Cramer‐Rao lower bounds; CV, coefficient of variation; Gln, glutamine; Glu, glutamate; GSH, glutathione; NAA, N‐acetylaspartate; NAAG, N‐acetylaspartylglutamate.

**FIGURE 7:**
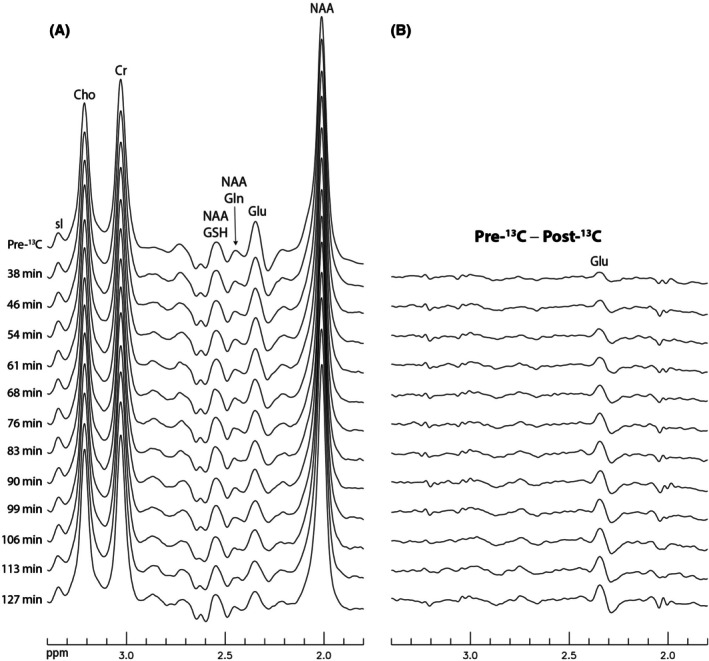
Time‐course ^1^H spectra and the differences between the pre‐^13^C spectrum and each post‐^13^C spectrum acquired from a single participant. (A) Pre‐^13^C spectrum and post‐^13^C spectra acquired at individual time points, each representing the sum of the corresponding ON and OFF spectra. (B) Differences between the pre‐^13^C spectrum and each post‐^13^C spectrum. Cho, choline; Cr, creatine; Gln, glutamine; Glu, glutamate; GSH, glutathione; NAA, N‐acetylaspartate.

Two of the 6 participants were scanned following oral administration of [U‐^13^C]glucose. Figure 7 shows a single participant's time‐course proton MRS spectra (Figure 7A) and the differences between the pre‐^13^C spectrum and each post‐^13^C spectrum (Figure 7B). These differences clearly demonstrated Glu turnover, as ^12^C at the parent Glu C4 position was replaced by ^13^C from the exogenous ^13^C‐labeled glucose.

## 4 DISCUSSION

This study aims to address the spectral overlap issues in the detection of Glu, Gln, and GSH at 3 T using an NAA‐CH_2_ difference editing pulse sequence combined with a novel TE optimization approach. Through difference editing, NAA‐CH_2_ is quantified independently of NAA‐CH_3_, thereby avoiding systematic errors that arise from forcing equal T_2_ decays for the two moieties. This, in turn, enhances the accuracy and reliability of Gln and GSH detection. The raw concentration ratio of NAA‐CH_2_ to NAA‐CH_3_, obtained directly from spectral fitting of the in vivo spectra, is 0.95 ± 0.03. This ratio is significantly below 1 (one‐sample, one‐tailed t‐test; *p* = 0.01), confirming that constrained fitting of the entire NAA molecule introduces systematic error in determining the NAA‐CH_2_ resonance signal. Because the NAA‐CH_3_ singlet signal is much stronger than the NAA‐CH_2_ signal, constrained fitting of the entire NAA molecule—which forces the concentrations of NAA‐CH_3_ and NAA‐CH_2_ to be equal—introduces only a small error (0.4%) in their absolute concentrations. However, the 5% error in the modeled NAA‐CH_2_ signal propagates to the detection of Gln and GSH. Monte Carlo analysis (Table 3) shows errors of 3.0% and 9.4% in Gln and GSH concentrations, respectively, which the proposed technique effectively eliminates.

The goal of the numerical TE optimization in this study is to minimize interference from the highly dominant Glu signal in the detection of Gln. This TE optimization is largely independent of the spectral editing of NAA‐CH_2_, as the editing pulse applied at 4.38 ppm avoids the resonances of Glu, Gln, and glutamyl‐GSH. A unique set of TE and TE_1_ values (TE = 85 ms, TE_1_ = 26 ms) is obtained through numerical optimization to minimize Glu interference in Gln detection. After subtracting the modeled NAA‐CH_2_ signal, along with the significantly smaller Asp and NAAG signals, the cleaned‐up in vivo sum spectrum revealed clearly separated Glu, Gln, and GSH peaks that closely resembled those from numerical simulations. Clear separation of spectral signals is considered highly important in clinical applications, as it enhances clarity, reduces ambiguity, lowers the risk of clinical errors, facilitates efficient interpretation, and supports clinical training and communication.^37^, ^38^ We believe this distinct spectral separation is important for clinical MRS studies, especially given recent findings of substantial quantification errors due to spectral overlap.^39^

The GABA level is not reported in Table 2, even though it is quantified in this study. In addition to the large uncertainty due to random noise, evidenced by its 24% ± 28% CRLB, the quantified GABA level is also prone to large errors. This is because the small GABA H2 peak at 2.28 ppm is overshadowed by the overlapping Glu H4 peak, and the GABA H4 peak at 3.01 ppm is completely obscured by the much larger Cr peak.

In addition to inverting the CH proton of NAA at 4.38 ppm, the editing pulse also perturbs the resonance signals of other metabolites. For example, the H2 proton of myo‐inositol at 4.05 ppm is slightly perturbed, influencing its coupled H1 and H3 protons at 3.52 ppm. Additionally, macromolecule resonances M_4.05–4.43_ ^40^ are perturbed, affecting their signals at 1.24 and 1.43 ppm through macromolecular J‐couplings. These affected resonances lie outside our fitting range of 1.8–3.4 ppm and therefore do not affect our quantification results.

Short‐TE PRESS sequences have been widely used in clinical MRS studies at 3 T for the in vivo detection of metabolites, including Glu, Gln, and GSH. MRS with a short TE reduces signal loss due to T_2_ decay but at the expense of strong macromolecule signals and severe spectral overlap among Glu, Gln, GSH, and NAA‐CH_2_ signals. These factors are known to introduce large uncertainties and systematic errors in the quantification of Glu, Gln, and GSH.^39^, ^41^ In particular, Zhang and Shen^39^ demonstrated that, due to severe spectral overlap, the Glu concentration computed by the commercial LCModel from short‐TE MRS data varies significantly when the linewidth of the same in vivo spectrum is slightly altered. Because many patients with neuropsychiatric disorders tend to make more head movements during relatively long MRS scans, the high sensitivity of Glu to spectral linewidth reported by short‐TE MRS is likely to introduce significant bias into clinical results. Therefore, minimizing spectral overlap is crucial for accurately determining Glu and other weaker metabolites, such as Gln and GSH.

As demonstrated in a previous study,^41^ Glu and Gln measurement at TE = 97 ms resulted in significantly lower CRLB values compared with TE = 35 ms. Numerical simulations in this study show that TE = 95 ms results in substantially greater Glu interference in Gln detection compared with TE = 85 ms (see Figure 4 and Table 1). Another study^16^ used a TE of 80 ms for in vivo detection of Glu at 3 T without modeling GSH. In contrast, GSH is fitted in this study after independently determining the overlapping NAA‐CH_2_ signal, without implicitly imposing a constraint of equal T_2_‐weighted concentrations for NAA‐CH_3_ and NAA‐CH_2_ in the spectral model. Furthermore, numerical simulations show that TE = 85 ms not only minimizes Glu interference in Gln detection but also yields higher peak heights for Glu and Gln compared with TE = 80 ms (see Table 1).

For the in vivo experiments following oral administration of [U‐^13^C]glucose, the difference spectra between the pre‐^13^C spectrum and each post‐^13^C spectrum clearly demonstrate the turnover of the spectrally resolved Glu. Because the turnover of spectrally resolved Glu is detected here using standard, commercially available hardware (a 3T scanner with a single proton channel and a commercially available proton head coil), the proposed method can be readily implemented on any commercial 3T scanner.

## 5 CONCLUSION

In this study, we introduced NAA‐CH_2_ difference editing combined with a novel TE optimization approach to achieve simultaneous and spectrally resolved detection of Glu, Gln, and GSH at 3 T. This technique enables accurate and reliable detection of NAA‐CH_2_ independently of NAA‐CH_3_, thereby eliminating systematic errors caused by the commonly used constrained fitting of the entire NAA molecule. Through numerical optimization, we determined an optimal set of TE and TE_1_ values (TE = 85 ms, TE_1_ = 26 ms) that minimized Glu interference in Gln detection. By subtracting the modeled NAA‐CH_2_, Asp, and NAAG signals, the resulting in vivo spectrum revealed well‐defined and spectrally resolved peaks for Glu, Gln, and GSH. Furthermore, the time‐course spectra following oral administration of [U‐^13^C]glucose demonstrated the feasibility of measuring ^13^C turnovers of spectrally resolved Glu at 3 T with the high sensitivity and spatial resolution of proton MRS.

## Supporting information


**Data S1.** Supporting information.

## Data Availability

The data acquired in this study and the code developed to analyze the acquired data are available at https://www.nitrc.org/doi/landing_page.php?doi=10.25790/bml0cm.178.
