# Supplementary material for: Simultaneous in vivo detection of spectrally resolved glutamate, glutamine, and glutathione at 3 T with NAA‐aspartyl editing and echo‐time optimization
Source: Magn Reson Med. 2025 Sep 8;95(2):668–79. doi: 10.1002/mrm.70076 (PMC12681290; doi:10.1002/mrm.70076)
Supplement: Supplementary file 1 — Data S1. Supporting information. [file MRM-95-668-s001.pdf]

Supporting information for

Simultaneous in vivo detection of spectrally resolved glutamate, glutamine, and glutathione at 3 T with NAA-aspartyl editing and echo-time optimization

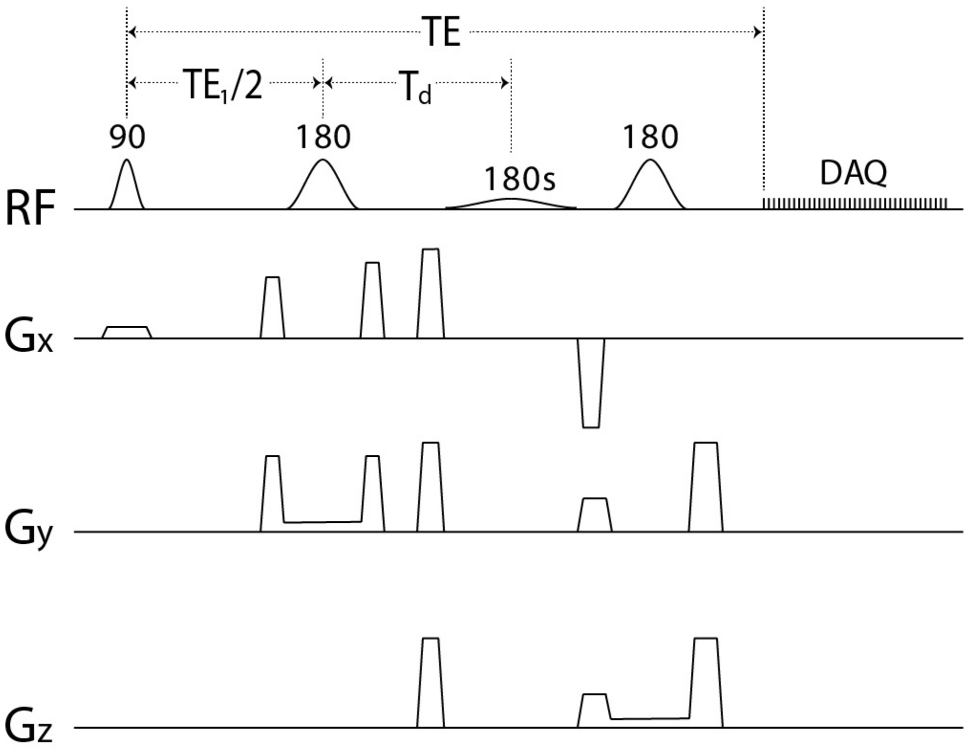

Figure S1. Schematic diagram of the NAA-CH_2 difference editing pulse sequence. The single custom-made editing pulse was applied at 4.38 ppm. TE = 85 ms; TE_1 = 26 ms; T_d = 21 ms. DAQ, data acquisition.

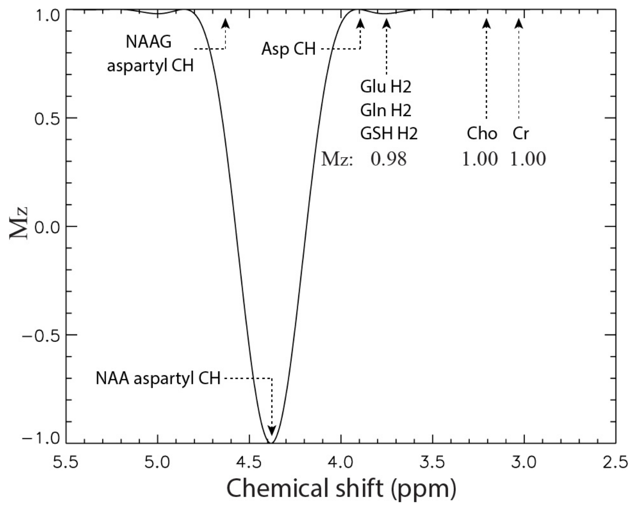

Figure S2. Frequency response of the editing pulse after removing the second band. M_z is expressed relative to the equilibrium magnetization M_0. This pulse was generated by setting the amplitude of the second Gaussian in the dual-band pulse to zero ($a_2 = 0$ in Table S1). Note the small but appreciable perturbation ($M_z = 0.98$) on the α-protons of Glu, Gln, and glutamyl-GSH.

Procedure S1: Generation of the Dual-Band Editing Pulse

The editing pulse was constructed using a superposition of two symmetrically truncated Gaussians with the same duration. The time-domain profile of an editing pulse can be expressed as:[23]

$$B_1(n) = \sum_{m=1}^{M} a_m[\exp(-(t_n w_m)^2/2) - \exp(-(t_N w_m)^2/2)] \exp[-i(\varphi_m + 2\pi\Delta\nu_m t_n T)]. \quad \text{(S1)}$$

The complex-valued $B_1(n)$, with $n = 1, 2, \ldots N$, represents the B_1 field of the nth point of the editing pulse, which comprises a total of N points. M denotes the number of truncated Gaussians, and a_m is the peak amplitude of the mth Gaussian. The unitless t_n represents the normalized time of the nth point of the editing pulse, given by $t_n = (n - 0.5) / N - 0.5$, with $t_N = 0.5 - 0.5 / N$. The unitless w_m is the full width of the mth Gaussian, expressed as a multiple of the standard deviation. $\Delta\nu_m$ denotes the frequency offset of the mth Gaussian from the frequency at which the editing pulse is

applied, and is given by $\Delta\nu_m = (ppm_m - ppm_{edit})B_0$, where ppm_m is the chemical shift of the mth Gaussian, ppm_{edit} is the chemical shift at which the editing pulse is applied, and B_0 is the scanner operating frequency in MHz. φ_m is the initial phase of the mth Gaussian, and T is the duration of the editing pulse. Specifically, the dual-band editing pulse used in this work comprised 400 points (N = 400), had a duration of 24 ms (T = 24 ms), and was constructed as the superposition of two truncated Gaussians (M = 2). Each Gaussian was defined by four parameters: ppm_m, a_m, φ_m, and w_m. These parameters were optimized using numerical calculations of the frequency response of the superimposed pulse based on the Bloch equations for each trial set of input parameters.[23] The optimized parameter values are given in Table S1. The editing pulse was applied at 4.38 ppm (ppm_{edit} = 4.38) with a nominal flip angle of 179.94°.

Table S1. Parameters for the dual-band editing pulse.

m	ppm_m	a_m (Hz)	φ_m	w_m
**1**	4.382	52.3	0.0	3.0
**2**	3.755	3.3	0.0	3.0
